# Some Observations on the Motions of the Mandible

**Published:** 1901-06-15

**Authors:** 


### BIBLIOGRAPHY.

"Some Observations on the Motions of the Mandible," by Charles Tomes.—The authors having referred to certain discussions upon the subject which have of late years been published, proceeded to describe the peculiarities of the temporo-mandibular articulation, drawing particular attention to the movements of the inter-articular cartilage and its relation to the condyle and the temporal bone, and showing that the resultant movement of the entire jaw is compounded of sliding and circular motions, and that the mandible in opening moves approximately in the arc of a circle.

The first step of their inquiry was the examination of the forms of the glenoid cavity and of the eminentia articularis in a number of skulls. The skulls referred to in the paper are those of the baboon, the howling monkey (Mycetes), the aye-aye, the chimpanzee, the orang and the gorilla; also a large number of human skulls ranging from those of Australian blacks to European skulls.

The authors found that the articular surface even in the highest monkeys has a far flatter surface than that of man. The strong curves of the human articulation have been gradually acquired. In man there are two conspicuous differences from even the highest of the apes, namely, the antero-posterior shortening of the jaw and the complete assumption of the upright position. Both of these factors tend to leave less room for the hyo-laryngeal apparatus when the mouth is widely opened. And the complex form of the articulation, which has the effect of carrying the jaw forward as it opens, gives so much more room, and in this way is an adaptation—hitherto it is believed unnoticed—to the upright position. In European skulls the variations in size and shape of the articular surface are very considerable, the size and shape of the condyles are even more variable, therefore the movements of the mandible must necessarily vary in different individuals.

The authors sum up their conclusions as follows:

1. The mandible in opening moves approximately in the arc of a circle.

2. The center of this circle is always below the level of the condyle, generally from an inch to an inch and a half. Its radius may be no greater than the length of the jaw, but commonly exceeds it, the center being usually considerally behind the condyle.

3. The path described is coincident with that of a simple rotation round a stationary condyle only at its start and its completion, and diverges forward as it descends.

4. The paths described in opening and in closing are never coincident, but the amount of their divergence is variable. The closure path usually lies in front ot the opening path.

5. The size and shape of the glenoid fossa and ot the eminentia articularis vary much in well developed European skulls; hence the paths described are not and can not be identical in different individuals.

6. While the form of the articulation stamps a certain similarity upon tracings taken from the same individual, the laxity of the ligaments and the varying pull of the different muscles allows of some latitude in the path pursued.

7. The form of the articular surface is in a measure peculiar to man, being most nearly approached by the gorilla and by the howling monkey.—*Dental Record.*

